# Cost-effectiveness analysis of biologics for the treatment of chronic rhinosinusitis with nasal polyps in Canada

**DOI:** 10.1186/s13223-023-00823-1

**Published:** 2023-10-14

**Authors:** Michael Yong, Keshinisuthan Kirubalingam, Martin Y. Desrosiers, Shaun J. Kilty, Andrew Thamboo

**Affiliations:** 1 Division of Otolaryngology, Head and Neck Surgery, University of British Columbia Faculty of Medicine, 1081 Burrard Street, Vancouver, BC V5Z 1Y6 Canada; 2 Faculty of Medicine, Queen’s University, Kingston, ON Canada; 3 Department of Otolaryngology, Centre de Recherche du Centre Hospitalier de L’Universite de Montreal, Montreal, QC Canada; 4 Department of Otolaryngology, Head and Neck Surgery, University of Ottawa, Ottawa, ON Canada

**Keywords:** Cost-effectiveness, Biologics, Chronic Rhinosinusitis, Nasal Polyps

## Abstract

**Background:**

Dupilumab, omalizumab, and mepolizumab are the three biologics currently approved for use in CRSwNP in Canada. Despite evidence of efficacy, their cost-effectiveness, which is a key factor influencing prescribing patterns, has not yet been compared to each other.

**Methods:**

A cost-effectiveness model using quality-adjusted life years (QALYs) was constructed using a Decision Tree Markov analysis. A third-party healthcare payer perspective and a 10-year time horizon was used. A willingness-to-pay (WTP) threshold of 50,000 Canadian dollars (CAD) per QALY was used to determine cost-effectiveness. Dupilumab, omalizumab, and mepolizumab were each compared to each other.

**Results:**

Omalizumab was the most cost-effective biologic using current estimates of cost and efficacy in CRSwNP. Using omalizumab as a baseline, dupilumab had an ICER of $235,305/QALY. Mepolizumab was dominated by omalizumab and dupilumab at the current drug prices and estimates of efficacy. Sensitivity analyses determined that when increasing the WTP threshold to $150,000/QALY, dupilumab became cost-effective compared to omalizumab in 22.5% of simulation scenarios. Additionally, altering dosing frequency had a significant effect on cost-effectiveness.

**Conclusion:**

When comparing the relative cost-effectiveness of biologics in recalcitrant CRSwNP, omalizumab currently appears to be the most cost-effective option. Future reductions in drug prices, adjustments to currently approved dosing regimens, better patient selection, and improvements in sinus surgery outcomes will challenge the current cost-effectiveness models and necessitate reassessment as treatments for CRSwNP continue to evolve.

## Introduction

Chronic rhinosinusitis (CRS) is characterized by concomitant inflammation of the nasal and sinus mucosa [1]. It can be distinguished into two phenotypes: chronic rhinosinusitis with nasal polys (CRSwNP) or chronic rhinosinusitis without nasal polyps (CRSsNP) [2]. CRSwNP is a leading cause of significant morbidity globally with estimated prevalence of 1–2.6% in the general population and 25–30% in patients with CRS [3, 4]. CRSwNP is a chronic inflammatory syndrome of the nasal passage linings or sinuses resulting in soft tissue growth known as nasal polyps [3]. As a Type 2 inflammatory disease, it is often associated with other conditions such as asthma, allergic rhinitis, and aspirin-exacerbated respiratory disease (AERD) [5]. Although medical therapy and endoscopic sinus surgery (ESS) have been the mainstay treatment options for patients with CRSwNP, there remains a significant population with limited response to currently available strategies [6].

The emergence of monoclonal antibodies targeting specific components of the Type 2 inflammatory pathway has catalyzed significant changes in the therapeutic landscape of CRSwNP treatment [7]. Recent clinical trial data has suggested that biologics can improve the clinical signs and symptoms of CRSwNP in patients with medically and/or surgically recalcitrant disease [8, 9]. There are currently three biologics approved for use in CRSwNP including dupliumab, omalizumab and mepolizumab in Canada. Though current evidence demonstrate higher efficacy in dupliumab users, emerging data suggest a promising role of omalizumab and mepolizumab in improving outcomes [10, 11]. Omalizumab and mepolizumab have been used in the treatment of asthma for over a decade with evidence indicating their efficacy [12]. However, despite the proven efficacy of biologics overall, the high cost of these pharmaceuticals has been cost-prohibitive, with recent cost-effectiveness analyses demonstrating their value principally in salvage treatment [13, 14]. Current Canadian practice guidelines on the use of biologics for treating CRSwNP reflect these findings, suggesting that biologics should only be considered in patients who have undergone adequate ESS and have failed appropriate medical therapy [15].

In Canada, most health systems are faced with high demand but have limited budget with which to provide the necessary services. Hence, the use of biologics in the most cost-efficient manner is important in promoting sustainability and healthcare stewardship. While there is now robust evidence detailing the comparative efficacy of each of the three biologics approved for use in Canada, there are no studies that compare the cost-effectiveness to each other. The aim of this study was to use all currently available evidence to perform a cost-effectiveness analysis comparing the relative cost-effectiveness of dupilumab, mepolizumab, and omalizumab for the treatment of CRSwNP.

## Methods

This was a cost-effectiveness analysis using reporting standards according to the 2013 Consolidated Health Economic Evaluation Reporting Standards (CHEERS) guidelines [16]. The population of interest consisted of adult patients with a diagnosis of CRSwNP which was refractory to initial ESS and appropriate medical management. The primary outcome in the analysis was the incremental cost per quality-adjusted life year (QALY), illustrated by the incremental cost-effectiveness ratio (ICER) for each treatment strategy. A third-party healthcare payer perspective was used and a 10-year time horizon was used. The ICER denotes how much payment is required for one additional year of (quality-weighted) life and is compared with a pre-determined “willingness-to-pay” (WTP) threshold that differs by publication, society, economic system, time period, and other factors [17]. A WTP threshold of 50,000 Canadian dollars (CAD) per QALY was used to determine cost-effectiveness. Discounting is a mathematical procedure for adjusting future costs and outcomes of health-care interventions to present value [18]. A discount rate of 3% was recommended by the Public Health Service Panel on Cost-Effectiveness in Medicine and has been used in other CEA analyses [13, 19]. Thus, an annual discount rate of 3% was applied to both costs and outcomes.

### Modeling approach

**Fig. 1:**
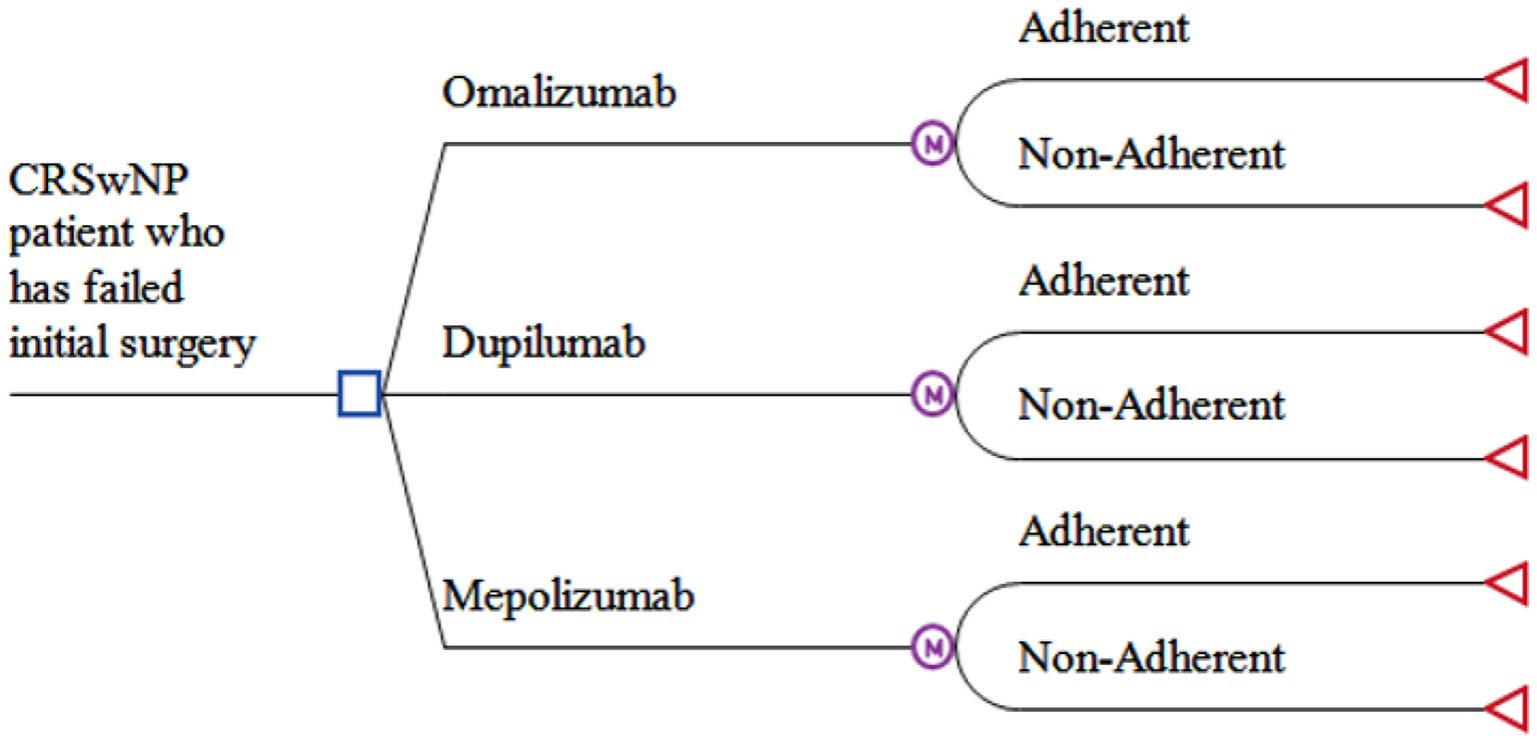
Markov decision tree analysis model—Model used to simulate the cost-effectiveness analysis

A decision tree and Markov model (Fig. 1) were constructed with the assistance of TreeAge Pro Healthcare Module 2015 (TreeAge Software, Inc., Williamstown, MA, USA). The status quo was revision ESS. This was directly compared against patients receiving either dupilumab, mepolizumab, or omalizumab. All data inputs for probabilities, utilities, and costs are presented in full in Table 1.

**Table 1:** Parameters used in model

Variable	Description	Mean	Standard deviation	Distribution	Source
Probabilities					
	Yearly probability of revision surgery on no biologic	0.03894*			[15]
	Yearly probability of revision surgery on dupilumab	0.00923	0.0022	Beta	[10]
	Yearly probability of revision surgery on mepolizumab	0.01686	0.0039	Beta	[10]
	Yearly probability of revision surgery on omalizumab	0.02592	0.0039	Beta	[10]
	Yearly probability of rescue OCS on no biologic	0.3196			[10]
	Yearly probability of rescue OCS on dupilumab	0.1023	0.0160	Beta	[10]
	Yearly probability of rescue OCS on mepolizumab	0.2173	0.0328	Beta	[10]
	Yearly probability of rescue OCS on omalizumab	0.1950	0.0911	Beta	[10]
	Yearly probability of adverse event on dupilumab	0.3183		Beta (2, 4.28)	[10]
	Yearly probability of adverse event on mepolizumab	0.3183		Beta (2, 4.28)	[10]
	Yearly probability of adverse event on omalizumab	0.3183		Beta (2, 4.28)	[10]
	Proportion of patients non-adherent to dupilumab	0.086		Beta (2, 21.2)	[11]
	Proportion of patients non-adherent to mepolizumab	0.112		Beta (2, 15.8)	[26]
	Proportion of patients non-adherent to omalizumab	0.052		Beta (2, 36)	[25]
Utilities					
	SNOT-22 of patients on no biologic	50.11			[10]
	SNOT-22 of patients on dupilumab	30.20	1.295	Beta	[10]
	SNOT-22 of patients on mepolizumab	37.22	1.848	Beta	[10]
	SNOT-22 of patients on omalizumab	34.02	1.895	Beta	[10]
Costs					
	Cost of ESS in Canada	$3,987			[36]
	Cost of biologic complication as anaphylaxis	$1,446			[28]
	Cost of biologic complication as arthritis	$3,562			[27]
	Cost of biologic complication as conjunctivitis	$179			[30]
	Cost of biologic complication as EGPA	$33,292			[29]
	Cost of biologic complication as hypersensitivity	$601			[28]
	Cost of biologic complication as injection reaction	$601			[28]
	Cost of dupilumab (average yearly)	$25,516			Product Monograph (Sanofi-Aventis Canada Inc.)
	Cost of mepolizumab (average yearly)	$27,383			Product Monograph (GlaxoSmithKline)
	Cost of omalizumab (average yearly)	$20,000			Drug Benefit Prices (Ontario Ministry of Health)

*OCS* Oral corticosteroid

*SNOT-22* Sinonasal Outcome Test 22-Item

*ESS* Endoscopic sinus surgery

*CMS* Centers for Medicare & Medicaid Services

*CPT* Current Procedural Terminology

*EGPA* Eosinophilic Granulomatosis with Polyangiitis

*Compounded yearly rate calculated from a 5-year rate of 10–30%

### Probabilities and outcomes

Probabilities in the analysis were extracted from published literature [10, 15, 20–26]. Briefly, database searches on PubMed and Embase were performed using the keywords “chronic rhinosinusitis”, “nasal polyps”, “biologics”, “outcome”, “dupilumab”, “omalizumab”, and “mepolizumab”. Articles examining the efficacy of dupilumab, omalizumab, and mepolizumab, as well as revision surgery, for the treatment of CRSwNP were reviewed. Systematic reviews and meta-analyses were prioritized as the source for model inputs. All model inputs can be found in Table 1.

The probability of complications from surgery followed a similar weighted calculation as previously published by Rudmik et al. [21, 27–30]. The probability and distribution of complications as a result of biologic therapy were considered to be the same for all biologics, according to data demonstrating no significant differences in adverse events between drugs [10]. The probability of requiring rescue oral corticosteroids and revision surgery were derived from the recent meta-analysis by Oykhman et al. [10]. A baseline yearly compounded rate of revision surgery on no biologic treatment was calculated to be 4% based on the literature-estimated 5-year revision surgery rate for patient with recalcitrant CRSwNP of 10–30%. [15]

Health utility was measured using QALYs converted from Sinonasal Outcome Test (SNOT-22) outcomes according to a previously published mapping algorithm [31]. SNOT-22 scores are the most common quality-of-life outcome measure used in evaluating the efficacy of biologics and surgical therapy for the treatment of CRSwNP.

### Costs

Costs associated with surgical and biologic therapy were derived from CPT codes from the Centers for Medicare & Medicaid Services (CMS) website, previously published literature data, and personal communication with pharmaceutical companies manufacturing with each biologic and pharmacy distributors. The yearly cost of biologic therapy was based on dosing regimens approved for use in Canada, which included every 2 weeks for dupilumab (total yearly cost of $25,516), every 4 weeks for mepolizumab (total yearly cost of $27,383), and every 4 weeks for omalizumab (total average yearly cost of $20,000) [32–35]. Omalizumab was priced at an average of 300 mg per dose reflecting recent trials. [25]

The mean (SD) cost of surgery was at $3,987, adjusted for inflation to 2021, from a Canadian estimate of the government-perspective costs of sinus surgery by Rudmik and Au [36]. All costs were reported in CAD and adjusted for inflation to 2021.

### Sensitivity analysis

Deterministic and probabilistic sensitivity analyses were performed to verify the robustness of the results. Deterministic sensitivity analysis explored threshold analysis and dosing frequency adjustment. The probabilistic sensitivity analysis used a Monte Carlo simulation with sufficient runs to generate stable ICER scatterplots and cost-acceptability curves, using beta distributions for probabilities and utilities. Standard deviations, where available, were derived from the literature.

## Results

**Fig. 2:**
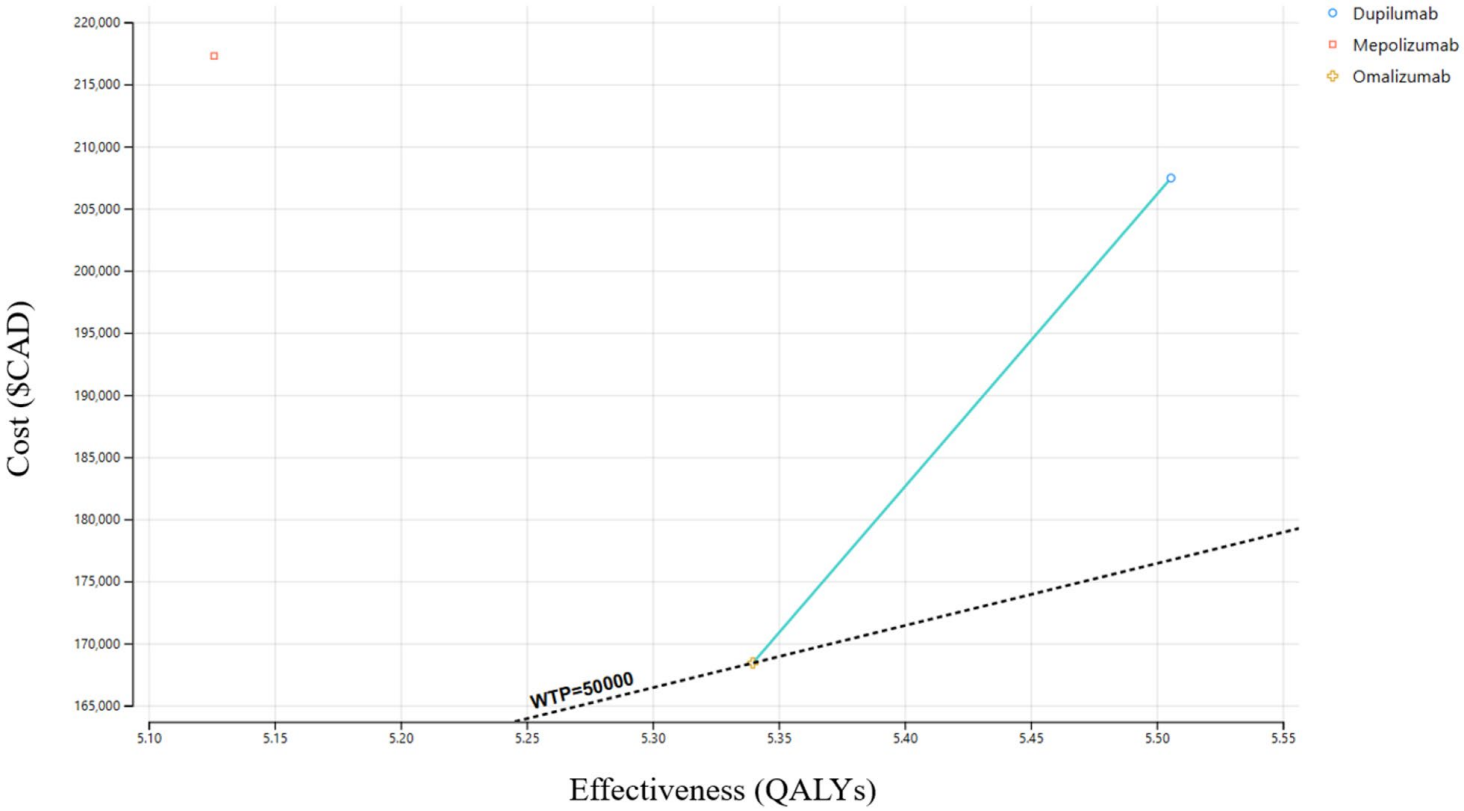
Cost-effectiveness plane—Cost-effectiveness plane comparing dupilumab, mepolizumab, and omalizumab to revision surgery

The cost-effectiveness base case analysis shows that omalizumab is currently the most cost-effective biologic for patients with CRSwNP who have persistent symptoms despite appropriate medical management and initial ESS, costing $168,414 over 10 years and accumulating 5.34 QALYs (Fig. 2). Using omalizumab as a baseline, dupilumab had an ICER of $235,305/QALY, costing $207,453 over 10 years and accumulating 5.51 QALYs (Table 2). Mepolizumab was dominated by omalizumab, costing $217,279 over 10 years and accumulating 5.13 QALYs (Table 2).

**Table 2:** Cost-effectiveness rankings of strategies

Strategy	Total costs	Total QALYs	ICER vs Omalizumab
1) Omalizumab	$168,414	5.34	–
2) Dupilumab	$207,453	5.51	$235,305/QALY
3) Mepolizumab	$217,280	5.13	Dominated by Omalizumab

### Sensitivity analysis

**Fig. 3:**
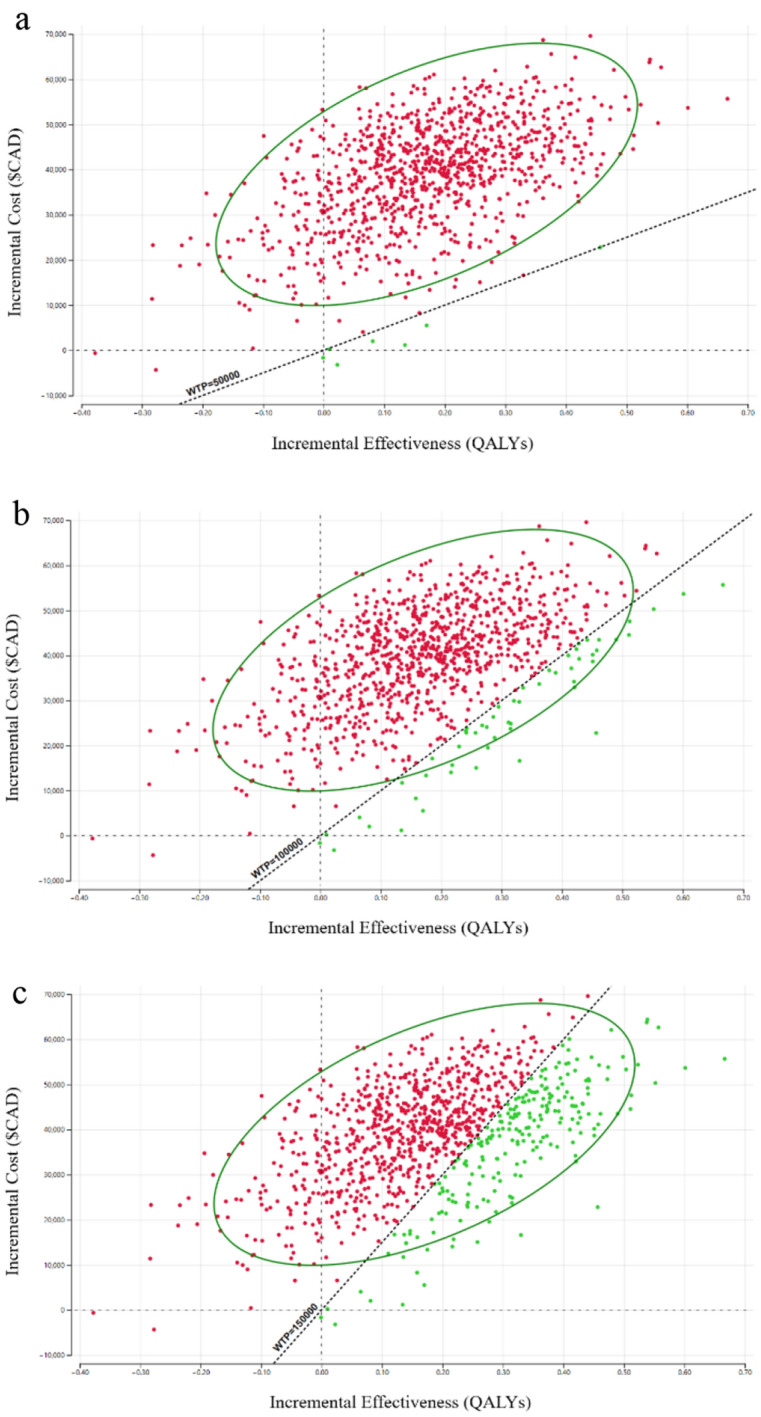
Probabilistic sensitivity analysis incremental cost-effectiveness scatterplot—Scatterplot showing the 95% confidence ellipse comparing the incremental cost-effectiveness of omalizumab compared to dupilumab at **a** Willingness-to-pay threshold of $50,000, **b** Willingness-to-pay threshold of $100,000, and **c** Willingness-to-pay threshold of $150,000

**Fig. 4:**
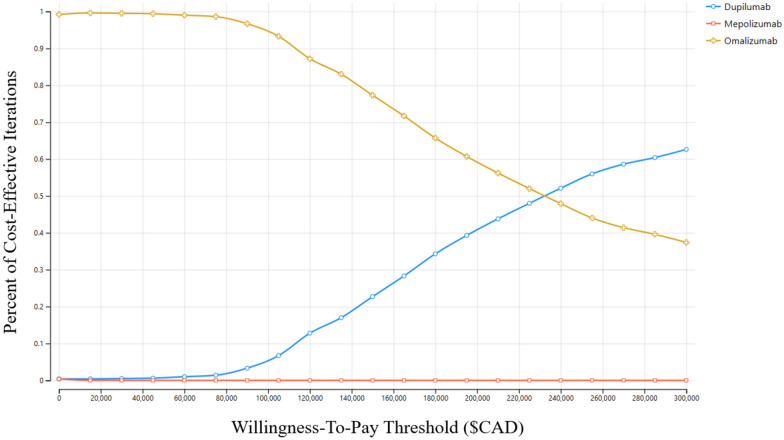
Probabilistic sensitivity analysis cost-acceptability curve—Cost-acceptability curve demonstrating the percentage of iterations that are cost-effective for each therapy based on various WTP thresholds

Cost-effectiveness between biologics was quite dependent on changes in cost and WTP threshold adjustments. Firstly, probabilistic sensitivity analysis showed that as the WTP threshold was increased to $100,000/QALY and to $150,000/QALY, the upper bounds of commonly accepted threshold in the United States, the percentage of iterations where dupilumab was cost-effective over omalizumab increased from 5% to 5.2% and 22.5%, respectively. These iterations are depicted in Fig. 3, showing the cost-effectiveness scatterplot and 95% confidence ellipse of dupilumab compared to omalizumab. Additionally, the cost-acceptability curve shown in Fig. 4 demonstrates that the WTP had to be raised past $230,000/QALY in order for dupilumab to become cost-effective compared to omalizumab in a majority of scenarios.

The one-way deterministic sensitivity analysis demonstrated the effect of varying the costs associated with each biologic. In order for dupilumab and mepolizumab to become cost-effective compared to omalizumab under the WTP threshold of $50,000/QALY, the cost of the cost of dupilumab would have to drop to $21,727 per year (-14.9%) and the cost of mepolizumab would have to drop to $19,855 per year (-27.5%). As an illustration of the effect of altered dosing regimens, if the assumed dosing regimen for dupilumab was reduced to once every month after the first 6 months of q2 weekly as in the LIBERTY NP SINUS-52 trial dosing and that the similar efficacy at 12 months post-treatment was maintained in perpetuity, dupilumab would dominate both omalizumab and mepolizumab, costing $125,187 per year and yielding 5.51 QALYs [37].

## Discussion

This analysis offers important insights into the cost-effectiveness of three biologics that are playing an increasingly important role in the treatment of CRSwNP in Canada. As the number of biologics approved for the treatment of recalcitrant CRSwNP continues to increase, it is essential that providers have additional data-driven guidance on the relative cost-effectiveness of these expensive medications within the context of currently accepted modalities of treatment such as ESS. Previous studies have demonstrated that biologics are not currently cost-effective when compared to surgery [13, 14]. However, while surgery is capable of treating nasal polyposis and sinonasal-related symptoms such as nasal obstruction, systemic medical therapy with biologics is currently the only means to altering the underlying inflammatory disorder.

When comparing the relative cost-effectiveness of the three biologics in this analysis, omalizumab was found to be more cost-effective than dupilumab, with mepolizumab being more costly and less effective than either omalizumab or dupilumab. These are important findings that need to be contextualized within recent literature supporting the superiority of dupilumab for the treatment of CRSwNP. A recent systematic review and meta-analysis has demonstrated that, using clinical trial data available to date, dupilumab demonstrates the greatest relative sinonasal-related quality of life improvements measured by mean SNOT-22 score improvement (-19.91 for dupilumab, -16.09 for omalizumab, and -12.89 for mepolizumab) [10]. This may lead some clinicians to favor dupilumab as the preferred initial biologic for CRSwNP patients [11]. However, the results of this cost-effectiveness analysis show that, when taking a health economics perspective, dupilumab does not always represent the optimal approach to treating CRSwNP and that other biologics should be considered as viable alternatives.

It is important to note that the sensitivity analysis demonstrated that if alternative and less frequent dosing regimens for biologics are approved for use, such as moving from every two week dosing to once monthly dosing for dupilumab which showed similar efficacy at 12 months post-treatment in the LIBERTY NP SINUS-52 trial by Bachert et al. the cost-effectiveness of biologics could shift in relation to each other [37]. Although the sensitivity analysis only explored decreasing the dosing frequency for dupilumab while keeping the price constant, this could potentially be an opportunity for other biologics as well. However, longer term follow-up data on alternative dosing regimens would be needed to support economic models which extrapolate efficacy during the lifetime of the treatment. Data surrounding less frequent dosing is currently being explored in further ongoing clinical studies, further paving the way for improved cost-effectiveness of biologics from reduced dosing frequency [38].

As guidelines continue to adapt in Canada based on the evidence available, there is an inclination in the guidelines to increase the cost-effectiveness of biologic drugs by targeting certain subgroups of patient populations, such as those patients with concomitant asthma [39]. With up to 67% of patients with CRSwNP having concomitant asthma, these patients have a higher sinonasal disease burden with severe symptoms and worse health-related quality of life along with higher associated healthcare costs, in addition to other quality of life impacts due to asthma itself [40–43]. While studies have shown that treatment of severe asthma with a biologic has resulted in improvements in asthma-related quality of life, it is possible that the usage of biologics for patients with CRSwNP and concomitant asthma would result in a synergistic improvement in quality of life and, thus, cost-effectiveness of any biologic for the treatment of these conditions together. It is also possible that the ICERs of each of dupilumab, omalizumab, and mepolizumab may change in this patient subgroup. For example, asthma literature has shown that mepolizumab achieves greater clinical improvements in asthma-related outcomes over omalizumab, which may not correlate with similar relative efficacy in CRSwNP [44–46]. Unfortunately, there is a current lack of data studying the overall quality of life gains that patients with CRSwNP and concomitant asthma might experience, as biologic outcomes for CRS and asthma have thus far been studied independently.

Considering that the single most important factor affecting the cost-effectiveness of any biologic in this analysis was the average yearly cost while on a biologic, current strategies to work towards improving overall cost-effectiveness should include a focus on reducing drug prices. Some of this work is currently underway, with future studies evaluating the efficacy of alternative dosing regimens which reduce the frequency of injections needed which, in turn, would reduce the overall price. Other approaches may include the establishment of preferential formulary status in return for lower prices and the development of benefit designs and negotiation platforms to provide a clear pathway for prescribers can help improve their use. Furthermore, clinicians can advocate for access to biologics through exceptional access programs in the subgroup of patients with recalcitrant CRSwNP. While biologics, for the time being, remain expensive and are not considered to be cost-effective when compared to ESS, clinicians are increasingly recognizing that each treatment modality, including topicals, surgery, and biologics, has a role in caring for patients with CRSwNP, and that tailoring treatment plans according to certain factors may help with this decision process.

### Limitations

A particular strength of the analysis in this paper comes from the use of data from a recently-published network meta-analysis synthesizing all available outcomes data on dupilumab, mepolizumab, and omalizumab [10]. As such, the estimates for health utility and other probability parameters used in this study can be considered to be robust and based on the highest-quality evidence currently available. However, there is currently a lack of data available from head-to-head trials between biologics comparing patient cohorts with similar disease severity, a limitation that introduces significant bias into the data that is currently available. Direct comparisons of current clinical trial data studying various biologics remains biased due to varying patient populations that were studied in each trial, including factors such as number of previous surgeries, nasal polyp scores, starting SNOT-22 scores, and proportion of patients with concomitant asthma or aspirin-exacerbated respiratory disease. Data from pending randomized clinical trials addressing these knowledge gaps will certainly lead to significant shifts in the relative cost-effectiveness of each biologic for the treatment of CRSwNP.

The difficulty of determining an accurate cost for each biologic medication and active research into alternative dosing regimens creates significant uncertainty is an inherent limitation of any cost-effectiveness analysis on biologics at the current moment. The concept of remission of the recalcitrant inflammatory state in patients with CRSwNP is a yet unexplored area of research, with recent data in asthma patients demonstrating that biologic therapy among a certain sub-population can result in long-term relief following cessation [47]. The use of biologics for a defined treatment duration in CRSwNP may have deep implications for reducing lifetime costs of these medications. Additionally, it is well-recognized that pharmaceutical companies may have publicly available list prices, but that these prices may not reflect the actual cost of the drug regimen that insurers eventually pay due to privately negotiated contracts that are not available for incorporation into cost-effectiveness models. Lastly, we recognize that the 10-year time horizon does not fully capture the true costs associated with a lifelong chronic condition such as chronic rhinosinusitis. However, due to the large amount of current research into alternative dosing regimens being conducted, it was felt that lengthening the time horizon any further would introduce a significant amount of uncertainty and further disadvantage biologics due to the assumption of use in perpetuity.

## Conclusion

Using current drug prices and efficacy estimates of dupilumab, omalizumab, and mepolizumab for the treatment of recalcitrant CRSwNP, omalizumab is the most cost-effective biologic. Sensitivity analysis showed that this conclusion was quite dependent on drug prices and dosing regimens. Additional data from randomized control trials, along with potential reductions in drug list prices, modification of dosing strategies, better patient selection, and improvements in sinus surgery outcomes, are all factors that are anticipated to shift the relative cost-effectiveness of biologic therapies and surgery, challenging current cost-effectiveness models and altering existing recommendations on biologic use in CRSwNP. As a consequence, these analyses should be reassessed over time as therapy and management of CRSwNP evolve.

## Data Availability

All study data are available in the Tables and Figures of this study. All other details not included in this publication are available upon request.

## Abbreviations

CEA: Cost effectiveness analysis
CRS: Chronic rhinosinusitis
CRSwNP: Chronic rhinosinusitis with nasal polyps
CRSsNP: Chronic rhinosinusitis without nasal polyps
AERD: Aspirin-exacerbated respiratory disease
QALY: Quality-adjusted life year
ESS: Endoscopic sinus surgery
ICER: Incremental cost-effectiveness ratio
CHEERS: Consolidated Health Economic Evaluation Reporting Standards
CMS: Centers for Medicare & Medicaid Services
CPT: Current procedural terminology
NADAC: National average drug acquisition cost
CPT: Current procedural terminology codes
CAD: Canadian dollar
WTP: Willingness-to-pay

## References

[1] Rudmik L, Smith TL (2011). Quality of life in patients with chronic rhinosinusitis. Curr Allergy Asthma Rep.

[2] Grayson JW, Cavada M, Harvey RJ (2019). Clinically relevant phenotypes in chronic rhinosinusitis. J Otolaryngol Head Neck Surg.

[3] Chen S, Zhou A, Emmanuel B, Thomas K, Guiang H (2020). Systematic literature review of the epidemiology and clinical burden of chronic rhinosinusitis with nasal polyposis. Curr Med Res Opin.

[4] Stevens WW, Schleimer RP, Kern RC (2016). Chronic rhinosinusitis with nasal polyps. J allergy Clin Immunol Pract.

[5] Stevens WW, Peters AT, Hirsch AG, Nordberg CM, Schwartz BS, Mercer DG (2017). Clinical characteristics of patients with chronic rhinosinusitis with nasal polyps, asthma, and aspirin-exacerbated respiratory disease. J allergy Clin Immunol Pract.

[6] DeConde AS, Mace JC, Levy JM, Rudmik L, Alt JA, Smith TL (2017). Prevalence of polyp recurrence after endoscopic sinus surgery for chronic rhinosinusitis with nasal polyposis. Laryngoscope.

[7] Ren L, Zhang N, Zhang L, Bachert C. Biologics for the treatment of chronic rhinosinusitis with nasal polyps—state of the art. World Allergy Organ J 2019;12(8). 10.1016/j.waojou.2019.100050 PMC6700446 31452831

[8] Patel GB, Peters AT (2021). The role of biologics in chronic rhinosinusitis with nasal polyps. Ear Nose Throat J.

[9] Fokkens WJ, Lund V, Bachert C, Mullol J, Bjermer L, Bousquet J (2019). EUFOREA consensus on biologics for CRSwNP with or without asthma. Allergy.

[10] Oykhman P, Paramo FA, Bousquet J, Kennedy DW, Brignardello-Petersen R, Chu DK (2022). Comparative efficacy and safety of monoclonal antibodies and aspirin desensitization for chronic rhinosinusitis with nasal polyposis: a systematic review and network meta-analysis. J Allergy Clin Immunol.

[11] Bachert C, Han JK, Desrosiers M, Hellings PW, Amin N, Lee SE (2019). Efficacy and safety of dupilumab in patients with severe chronic rhinosinusitis with nasal polyps (LIBERTY NP SINUS-24 and LIBERTY NP SINUS-52): results from two multicentre, randomised, double-blind, placebo-controlled, parallel-group phase 3 trials. Lancet (London, England)..

[12] Giovannini M, Mori F, Barni S, De Martino M, Novembre E (2019). Omalizumab and mepolizumab in the landscape of biological therapy for severe asthma in children: how to choose?. Ital J Pediatr.

[13] Yong M, Wu YQ, Howlett J, Ballreich J, Walgama E, Thamboo A (2021). Cost-effectiveness analysis comparing dupilumab and aspirin desensitization therapy for chronic rhinosinusitis with nasal polyposis in aspirin-exacerbated respiratory disease. Int Forum Allergy Rhinol.

[14] Scangas GA, Wu AW, Ting JY, Metson R, Walgama E, Shrime MG (2021). Cost utility analysis of dupilumab versus endoscopic sinus surgery for chronic rhinosinusitis with nasal polyps. Laryngoscope.

[15] Thamboo A, Kilty S, Witterick I, Chan Y, Chin CJ, Janjua A (2021). Canadian Rhinology Working Group consensus statement: biologic therapies for chronic rhinosinusitis. J Otolaryngol Head Neck Surg.

[16] Husereau D, Drummond M, Petrou S, Carswell C, Moher D, Greenberg D (2013). Consolidated health economic evaluation reporting standards (CHEERS) statement. BMJ.

[17] Verma V, Sprave T, Haque W, Simone CB, Chang JY, Welsh JW (2018). A systematic review of the cost and cost-effectiveness studies of immune checkpoint inhibitors. J Immunother Cancer.

[18] Attema AE, Brouwer WBF, Claxton K (2018). Discounting in Economic Evaluations. Pharmacoeconomics.

[19] Siegel JE, Weinstein MC, Russell LB, Gold MR (1996). Recommendations for reporting cost-effectiveness analyses. J Am Med Assoc.

[20] Dalziel K, Stein K, Round A, Garside R, Royle P (2006). Endoscopic sinus surgery for the excision of nasal polyps: a systematic review of safety and effectiveness. Am J Rhinol.

[21] Rudmik L, Soler ZM, Mace JC, Schlosser RJ, Smith TL (2015). Economic evaluation of endoscopic sinus surgery versus continued medical therapy for refractory chronic rhinosinusitis. Laryngoscope.

[22] Stankiewicz JA, Lal D, Connor M, Welch K (2011). Complications in endoscopic sinus surgery for chronic rhinosinusitis: a 25-year experience. Laryngoscope.

[23] Lindstrom DR, Toohill RJ, Loehrl TA, Smith TL (2004). Management of cerebrospinal fluid rhinorrhea: the Medical College of Wisconsin experience. Laryngoscope.

[24] Ramakrishnan VR, Kingdom TT, Nayak JV, Hwang PH, Orlandi RR (2012). Nationwide incidence of major complications in endoscopic sinus surgery. Int Forum Allergy Rhinol.

[25] Gevaert P, Omachi TA, Corren J, Mullol J, Han J, Lee SE (2020). Efficacy and safety of omalizumab in nasal polyposis: 2 randomized phase 3 trials. J Allergy Clin Immunol.

[26] Han JK, Bachert C, Fokkens W, Desrosiers M, Wagenmann M, Lee SE (2021). Mepolizumab for chronic rhinosinusitis with nasal polyps (SYNAPSE): a randomised, double-blind, placebo-controlled, phase 3 trial. Lancet Respir Med.

[27] Murphy LB, Cisternas MG, Pasta DJ, Helmick CG, Yelin EH. Medical expenditures and earnings losses among US adults with arthritis in 2013. Arthritis Care Res [Internet]. 2018;70(6):869–76. https://pubmed-ncbi-nlm-nih-gov.proxy1.library.jhu.edu/28950426/. 10.1002/acr.23425 28950426

[28] Patel DA, Holdford DA, Edwards E, Carroll N V. Estimating the economic burden of food-induced allergic reactions and anaphylaxis in the United States. J Allergy Clin Immunol [Internet]. 2011;128(1):110–115.e5. https://pubmed-ncbi-nlm-nih-gov.proxy1.library.jhu.edu/21489610/. 10.1016/j.jaci.2011.03.013 21489610

[29] Bell CF, Lau M. Clinical and economic characteristics of patients diagnosed with eosinophilic granulomatosis with polyangiitis (EGPA, formerly churg-strauss syndrome) in the United States. Arthritis Rheumatol [Internet]. 2018;70:1949–50. http://ovidsp.ovid.com/ovidweb.cgi?T=JS&PAGE=reference&D=emexa&NEWS=N&AN=626434784.

[30] Smith AF, Waycaster C. Estimate of the direct and indirect annual cost of bacterial conjunctivitis in the United States. BMC Ophthalmol [Internet]. 2009;9(1). https://pubmed-ncbi-nlm-nihgov.proxy1.library.jhu.edu/19939250/. 10.1186/1471-2415-9-13 PMC2791746 19939250

[31] Crump RT, Lai E, Liu G, Janjua A, Sutherland JM (2017). Establishing utility values for the 22-item Sino-Nasal Outcome Test (SNOT-22) using a crosswalk to the EuroQol–five-dimensional questionnaire–three-level version (EQ-5D-3L). Int Forum Allergy Rhinol.

[32] Drug Benefit Prices (DBPs) for products reimbursed under the EAP [Internet]. Ontario Ministry of Health. https://health.gov.on.ca/en/pro/programs/drugs/odbf/odbf_except_access.aspx. Accessed 30 May 2022.

[33] Nucala (mepolizumab). Product Monograph [Internet]. GlaxoSmithKline. 2021. https://health-products.canada.ca/dpd-bdpp/index-eng.jsp. Accessed 30 May 2022.

[34] Dupixent (dupilumab). Product Monograph [Internet]. Snofi-Aventis Canada Inc. 2022. https://health-products.canada.ca/dpd-bdpp/index-eng.jsp. Accessed 30 May 2022.

[35] Xolair (omalizumab). Product Monograph [Internet]. Novartis Pharma Canada. 2021 https://health-products.canada.ca/dpd-bdpp/index-eng.jsp. Accessed 30 May 2022.

[36] Au J, Rudmik L (2013). Cost of outpatient endoscopic sinus surgery from the perspective of the Canadian government: a time-driven activity-based costing approach. Int Forum Allergy Rhinol..

[37] Bachert C, Han JK, Desrosiers M, Hellings PW, Amin N, Lee SE (2019). Efficacy and safety of dupilumab in patients with severe chronic rhinosinusitis with nasal polyps (LIBERTY NP SINUS-24 and LIBERTY NP SINUS-52): results from two multicentre, randomised, double-blind, placebo-controlled, parallel-group phase 3 trials. Lancet (London, England).

[38] Efficacy and Safety of Depemokimab (GSK3511294) in participants with chronic rhinosinusitis with nasal polyps - full text view—clinicaltrials.gov [Internet]. https://www.clinicaltrials.gov/ct2/show/NCT05274750. Accessed 10 Aug 2022.

[39] McQueen RB, Sheehan DN, Whittington MD, van Boven JFM, Campbell JD (2018). Cost-effectiveness of biological asthma treatments: a systematic review and recommendations for future economic evaluations. Pharmacoeconomics.

[40] Alobid I, Cardelus S, Benítez P, Guilemany JM, Roca-Ferrer J, Picado C (2011). Persistent asthma has an accumulative impact on the loss of smell in patients with nasal polyposis. Rhinology.

[41] Bhattacharyya N, Villeneuve S, Joish VN, Amand C, Mannent L, Amin N (2019). Cost burden and resource utilization in patients with chronic rhinosinusitis and nasal polyps. Laryngoscope.

[42] Scangas GA, Remenschneider AK, Su BM, Shrime MG, Metson R (2017). The impact of asthma on the cost effectiveness of surgery for chronic rhinosinusitis with nasal polyps. Int Forum Allergy Rhinol..

[43] Wang M, Bu X, Luan G, Lin L, Wang Y, Jin J (2020). Distinct type 2-high inflammation associated molecular signatures of chronic rhinosinusitis with nasal polyps with comorbid asthma. Clin Transl Allergy..

[44] Claudia C, Campisi R, Nolasco S, Heffler E, Valenti G, Pelaia C (2020). Omalizumab versus mepolizumab in severe asthma: a propensity score matched efficiency retrospective cohort study. Eur Respir J.

[45] Chapman KR, Albers FC, Chipps B, Muñoz X, Devouassoux G, Bergna M (2019). The clinical benefit of mepolizumab replacing omalizumab in uncontrolled severe eosinophilic asthma. Allergy.

[46] Otto A, Dyer A, Smith B, Gupta R, Stynes G, Cockle S (2016). Comparative effectiveness of mepolizumab and omalizumab in severe asthma: an indirect comparison. J Allergy Clin Immunol.

[47] Menzies-Gow A, Hoyte FL, Price DB, Cohen D, Barker P, Kreindler J (2022). Clinical remission in severe asthma: a pooled post hoc analysis of the patient journey with benralizumab. Adv Ther.

